# 1-(3,4-Di­fluoro­benz­yl)-4-(4-methyl­phenyl­sulfon­yl)piperazine

**DOI:** 10.1107/S1600536813016462

**Published:** 2013-06-29

**Authors:** S. Sreenivasa, H. C. Anitha, P. A. Suchetan, B. S. Palakshamurthy, J. Savanur, J. Tonannavar

**Affiliations:** a Department of Studies and Research in Chemistry, Tumkur University, Tumkur Karnataka 572 103, India; b Center for Advanced Materials and Department of Chemistry, Tumkur University, Tumkur Karnataka, 572 103, India; c Department of Studies and Research in Physics, U.C.S. Tumkur University, Tumkur Karnataka 572 103, India; d Department of Physics, Karnatak University, Dharwad, Karnataka 580 003, India

## Abstract

In the title compound, C_18_H_20_F_2_N_2_O_2_S, the central piperazine ring adopts a chair conformation. The dihedral angle between the two benzene rings is 40.20°, whereas those between the piperazine ring (considering the best fit plane through all the non-H atoms) and the sulfonyl-bound benzene and di­fluoro­benzene rings are 74.96 and 86.16°, respectively. In the crystal, mol­ecules are stacked along the *a* axis through weak C—H⋯O and C—H⋯F inter­actions.

## Related literature

For similar structures, see: Sreenivasa *et al.* (2013*a* ▶,*b* ▶,*c* ▶).
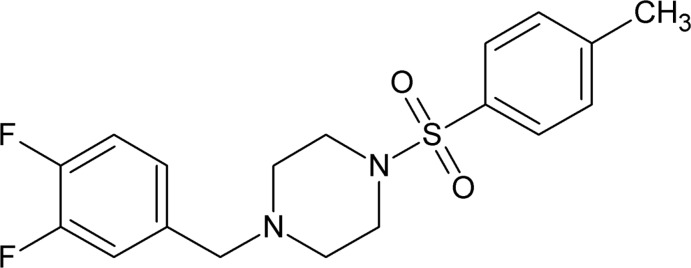


## Experimental

### 

#### Crystal data

C_18_H_20_F_2_N_2_O_2_S
*M* *_r_* = 366.42
Monoclinic, 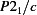

*a* = 6.6680 (2) Å
*b* = 36.0404 (8) Å
*c* = 7.6093 (2) Å
β = 99.728 (2)°
*V* = 1802.35 (8) Å^3^
*Z* = 4
Mo *K*α radiation
μ = 0.21 mm^−1^
*T* = 298 K
0.28 × 0.24 × 0.20 mm

#### Data collection

Bruker APEXII diffractometer
Absorption correction: multi-scan (*SADABS*; Bruker, 2009 ▶) *T*_min_ = 0.943, *T*_max_ = 0.959
9583 measured reflections
2434 independent reflections
1910 reflections with *I* > 2σ(*I*)
*R*_int_ = 0.025
θ_max_ = 22.8°

#### Refinement

*R*[*F* ^2^ > 2σ(*F* ^2^)] = 0.045
*wR*(*F* ^2^) = 0.114
*S* = 1.02
2434 reflections
227 parameters
H-atom parameters constrained
Δρ_max_ = 0.19 e Å^−3^
Δρ_min_ = −0.25 e Å^−3^

### 

Data collection: *APEX2* (Bruker, 2009 ▶); cell refinement: *APEX2* and *SAINT-Plus* (Bruker, 2009 ▶); data reduction: *SAINT-Plus* and *XPREP* (Bruker, 2009 ▶); program(s) used to solve structure: *SHELXS97* (Sheldrick, 2008 ▶); program(s) used to refine structure: *SHELXL97* (Sheldrick, 2008 ▶); molecular graphics: *Mercury* (Macrae *et al.*, 2008 ▶); software used to prepare material for publication: *SHELXL97*.

## Supplementary Material

Crystal structure: contains datablock(s) I, global. DOI: 10.1107/S1600536813016462/sj5330sup1.cif

Structure factors: contains datablock(s) I. DOI: 10.1107/S1600536813016462/sj5330Isup2.hkl

Supplementary material file. DOI: 10.1107/S1600536813016462/sj5330Isup3.cml

Additional supplementary materials:  crystallographic information; 3D view; checkCIF report

## Figures and Tables

**Table 1:** Hydrogen-bond geometry (Å, °)

*D*—H⋯*A*	*D*—H	H⋯*A*	*D*⋯*A*	*D*—H⋯*A*
C3—H3⋯O1^i^	0.93	2.67	3.380 (4)	134
C7—H7*A*⋯O1^ii^	0.96	2.66	3.400 (4)	134
C10—H10*B*⋯F1^iii^	0.97	2.66	3.585 (3)	160

Symmetry codes: (i) 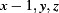; (ii) 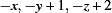; (iii) 
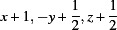
.

**Fig. 1.**
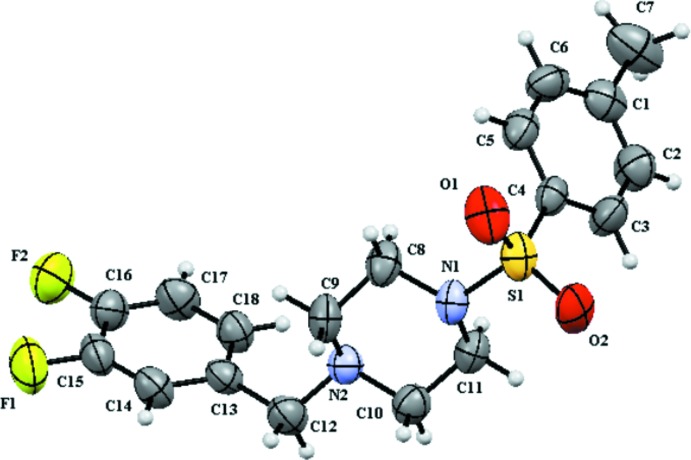
Molecular structure of the title compound, showing the atom-labeling scheme. Displacement ellipsoids are drawn at the 50% probability level.

**Fig. 2.**
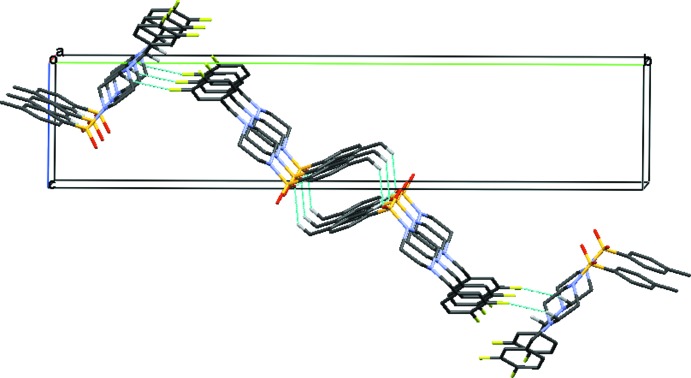
A packing diagram of the title compound, showing C–H···O and C–H···F interactions (dotted lines). Hydrogen atoms not involved in hydrogen bonding are omitted.

## supplementary crystallographic information

### Comment

As a part of our continued efforts to study the crystal structures of *N*-(aryl)(4-tosylpiperazin-1-yl)methanone derivatives (Sreenivasa *et al.*, 2013*a*,**b*,c*), we report herein the crystal structure of the title compound.

The title compound, Fig. 1, crystallizes in the monoclinic crystal system and *P*2_1_/*c* space group. The piperazine ring in the title compound adopts a chair conformation. The dihedral angle between the two benzene rings is 40.20°, compared to the observed dihedral angles of 72.2 (12)°, 76.86° and 30.97 (2)° respectively in (I), 1-(2,4-dichlorobenzyl)-4-[(4-methyl-phenyl)sulfonyl]-piperazine (Sreenivasa *et al.*, 2013*a*), (II) 1-tosyl-4-[2-(trifluoromethyl)-benzyl]piperazine (Sreenivasa *et al.*, 2013*b*) and (III) (2,3-difluorophenyl)(4-tosylpiperazin-1-yl)methanone (Sreenivasa *et al.*, 2013*c*). Further, the dihedral angles between the piperazine ring (considering the best fit plane through all the non-hydrogen atoms) and the sulfonyl bound benzene and difluorobenzene rings are 74.96° and 86.16° respectively, compared to 74.16 (2)° and 2.44 (13)° in I, 74.36° and 68.29 (3)° in II, and 69.4 (2)° and 75.98 (2)° in III.

In the crystal structure, the molecules are stacked along the *a* axis through weak C–H···O and C–H···F interactions, Fig. 2.

### Experimental

A mixture of 1-tosylpiperazine (0.01 mmol), potassium carbonate (0.03 mmol) and 3,4-difluorobenzyl bromide (0.01 mmol) was added to dry acetonitrile (5 ml). The mixture was stirred at 85°C for 8 h. The reaction was monitored by TLC. Solvent was removed by vacuum distillation and the crude product obtained was purified by column chromatography using 230–400 silica gel and petroleum ether/ethyl acetate as eluent.

Colourless prisms were obtained from a mixture of dichloromethane/methanol (7:3) by slow evaporation.

### Refinement

H atoms were positioned with idealized geometry using a riding model with C—H = 0.93 - 0.96 Å. The isotropic displacement parameters for all H atoms were set to 1.2 times *U*_eq_ of the parent atom or 1.5 times that of the parent atom for CH_3_.

Crystals were small and very weakly diffracting, with no significant data obtained beyond θ = 22.8° hence the low values of sin(θ/λ). However the structure solved and refined satisfactorily and gave acceptable residuals and su values.

### Crystal data

**Table:**

C_18_H_20_F_2_N_2_O_2_S	prism
*M**_r_* = 366.42	*D*_x_ = 1.350 Mg m^−^^3^
Monoclinic, *P*2_1_/*c*	Melting point: 501 K
Hall symbol: -P 2ybc	Mo *K*α radiation, λ = 0.71073 Å
*a* = 6.6680 (2) Å	Cell parameters from 227 reflections
*b* = 36.0404 (8) Å	θ = 2.3–22.8°
*c* = 7.6093 (2) Å	µ = 0.21 mm^−^^1^
β = 99.728 (2)°	*T* = 298 K
*V* = 1802.35 (8) Å^3^	Prism, colourless
*Z* = 4	0.28 × 0.24 × 0.20 mm
*F*(000) = 768	

### Data collection

**Table:**

Bruker APEXII diffractometer	2434 independent reflections
Radiation source: fine-focus sealed tube	1910 reflections with *I* > 2σ(*I*)
Graphite monochromator	*R*_int_ = 0.025
Detector resolution: 1.03 pixels mm^-1^	θ_max_ = 22.8°, θ_min_ = 2.3°
φ and ω scans	*h* = −7→7
Absorption correction: multi-scan (*SADABS*; Bruker, 2009)	*k* = −39→37
*T*_min_ = 0.943, *T*_max_ = 0.959	*l* = −7→8
9583 measured reflections	

### Refinement

**Table:**

Refinement on *F*^2^	Primary atom site location: structure-invariant direct methods
Least-squares matrix: full	Secondary atom site location: difference Fourier map
*R*[*F*^2^ > 2σ(*F*^2^)] = 0.045	Hydrogen site location: inferred from neighbouring sites
*wR*(*F*^2^) = 0.114	H-atom parameters constrained
*S* = 1.02	*w* = 1/[σ^2^(*F*_o_^2^) + (0.0512*P*)^2^ + 0.6421*P*] where *P* = (*F*_o_^2^ + 2*F*_c_^2^)/3
2434 reflections	(Δ/σ)_max_ = 0.028
227 parameters	Δρ_max_ = 0.19 e Å^−^^3^
0 restraints	Δρ_min_ = −0.25 e Å^−^^3^
32 constraints	

### Special details

**Table:**

Geometry. All e.s.d.'s (except the e.s.d. in the dihedral angle between two l.s. planes) are estimated using the full covariance matrix. The cell e.s.d.'s are taken into account individually in the estimation of e.s.d.'s in distances, angles and torsion angles; correlations between e.s.d.'s in cell parameters are only used when they are defined by crystal symmetry. An approximate (isotropic) treatment of cell e.s.d.'s is used for estimating e.s.d.'s involving l.s. planes.
Refinement. Refinement of *F*^2^ against ALL reflections. The weighted *R*-factor *wR* and goodness of fit *S* are based on *F*^2^, conventional *R*-factors *R* are based on *F*, with *F* set to zero for negative *F*^2^. The threshold expression of *F*^2^ > σ(*F*^2^) is used only for calculating *R*-factors(gt) *etc*. and is not relevant to the choice of reflections for refinement. *R*-factors based on *F*^2^ are statistically about twice as large as those based on *F*, and *R*- factors based on ALL data will be even larger.

### Fractional atomic coordinates and isotropic or equivalent isotropic displacement parameters (Å^2^)

**Table:**

	*x*	*y*	*z*	*U*_iso_*/*U*_eq_	
C1	−0.3654 (5)	0.51803 (9)	0.7226 (4)	0.0763 (8)	
C2	−0.4516 (5)	0.48860 (11)	0.7956 (4)	0.0850 (9)	
H2	−0.5880	0.4901	0.8070	0.102*	
C3	−0.3451 (5)	0.45692 (9)	0.8529 (4)	0.0761 (8)	
H3	−0.4088	0.4376	0.9028	0.091*	
C4	−0.1454 (4)	0.45409 (7)	0.8359 (3)	0.0598 (7)	
C5	−0.0564 (4)	0.48283 (9)	0.7599 (4)	0.0821 (9)	
H5	0.0790	0.4811	0.7455	0.099*	
C6	−0.1666 (6)	0.51438 (9)	0.7045 (4)	0.0870 (9)	
H6	−0.1034	0.5336	0.6535	0.104*	
C7	−0.4821 (6)	0.55287 (10)	0.6662 (5)	0.1135 (13)	
H7A	−0.4283	0.5729	0.7431	0.170*	
H7B	−0.6229	0.5492	0.6742	0.170*	
H7C	−0.4697	0.5587	0.5455	0.170*	
C8	−0.2454 (4)	0.36712 (8)	0.6955 (4)	0.0737 (8)	
H8A	−0.3419	0.3850	0.6347	0.088*	
H8B	−0.2926	0.3592	0.8033	0.088*	
C9	−0.2310 (5)	0.33415 (7)	0.5761 (4)	0.0738 (8)	
H9A	−0.1406	0.3157	0.6400	0.089*	
H9B	−0.3644	0.3230	0.5431	0.089*	
C10	0.0484 (4)	0.36109 (8)	0.4655 (4)	0.0734 (8)	
H10A	0.1008	0.3680	0.3587	0.088*	
H10B	0.1392	0.3427	0.5292	0.088*	
C11	0.0404 (4)	0.39451 (7)	0.5809 (4)	0.0680 (7)	
H11A	0.1762	0.4046	0.6154	0.082*	
H11B	−0.0443	0.4134	0.5148	0.082*	
C12	−0.1536 (5)	0.31498 (8)	0.2910 (4)	0.0801 (9)	
H12A	−0.0854	0.2938	0.3529	0.096*	
H12B	−0.0773	0.3224	0.1990	0.096*	
C13	−0.3660 (4)	0.30386 (8)	0.2051 (3)	0.0629 (7)	
C14	−0.4280 (5)	0.26741 (8)	0.2015 (4)	0.0737 (8)	
H14	−0.3405	0.2492	0.2572	0.088*	
C15	−0.6186 (5)	0.25787 (8)	0.1158 (4)	0.0759 (8)	
C16	−0.7479 (5)	0.28422 (10)	0.0346 (4)	0.0772 (8)	
C17	−0.6903 (5)	0.32020 (9)	0.0356 (4)	0.0834 (9)	
H17	−0.7788	0.3381	−0.0212	0.100*	
C18	−0.4993 (5)	0.33006 (8)	0.1215 (4)	0.0729 (8)	
H18	−0.4596	0.3548	0.1231	0.088*	
N1	−0.0432 (3)	0.38434 (6)	0.7410 (3)	0.0646 (6)	
N2	−0.1548 (3)	0.34542 (6)	0.4167 (3)	0.0627 (6)	
O1	0.2056 (3)	0.42331 (6)	0.9324 (3)	0.0918 (7)	
O2	−0.0916 (4)	0.39768 (6)	1.0463 (2)	0.0989 (7)	
F1	−0.6798 (3)	0.22224 (5)	0.1108 (3)	0.1225 (8)	
F2	−0.9357 (3)	0.27392 (6)	−0.0489 (3)	0.1200 (7)	
S1	−0.00612 (12)	0.41390 (2)	0.90583 (9)	0.0734 (3)	

### Atomic displacement parameters (Å^2^)

**Table:**

	*U*^11^	*U*^22^	*U*^33^	*U*^12^	*U*^13^	*U*^23^
C1	0.080 (2)	0.089 (2)	0.0536 (17)	0.0093 (19)	−0.0063 (15)	−0.0117 (15)
C2	0.0544 (19)	0.114 (3)	0.086 (2)	0.001 (2)	0.0093 (16)	−0.022 (2)
C3	0.074 (2)	0.087 (2)	0.0703 (19)	−0.0205 (18)	0.0190 (15)	−0.0075 (16)
C4	0.0567 (18)	0.0677 (18)	0.0527 (15)	−0.0136 (13)	0.0024 (12)	−0.0043 (13)
C5	0.0567 (18)	0.077 (2)	0.114 (3)	−0.0079 (16)	0.0178 (17)	0.0131 (18)
C6	0.092 (3)	0.071 (2)	0.099 (2)	−0.0020 (18)	0.0179 (19)	0.0170 (17)
C7	0.128 (3)	0.116 (3)	0.082 (2)	0.047 (2)	−0.023 (2)	−0.014 (2)
C8	0.082 (2)	0.079 (2)	0.0601 (17)	−0.0242 (16)	0.0110 (15)	0.0036 (15)
C9	0.084 (2)	0.0610 (17)	0.0711 (19)	−0.0216 (15)	−0.0014 (15)	0.0047 (14)
C10	0.0626 (19)	0.076 (2)	0.0795 (19)	−0.0024 (15)	0.0056 (14)	0.0011 (16)
C11	0.0652 (18)	0.0661 (18)	0.0705 (18)	−0.0125 (14)	0.0051 (14)	0.0079 (14)
C12	0.073 (2)	0.077 (2)	0.086 (2)	0.0074 (16)	0.0020 (16)	−0.0156 (17)
C13	0.0687 (19)	0.0607 (18)	0.0582 (16)	0.0072 (15)	0.0069 (13)	−0.0111 (13)
C14	0.086 (2)	0.0618 (19)	0.0689 (18)	0.0114 (16)	0.0011 (16)	−0.0066 (14)
C15	0.093 (2)	0.0558 (19)	0.080 (2)	−0.0109 (18)	0.0167 (18)	−0.0134 (15)
C16	0.066 (2)	0.087 (2)	0.075 (2)	−0.0040 (18)	−0.0003 (15)	−0.0177 (17)
C17	0.082 (2)	0.079 (2)	0.083 (2)	0.0152 (18)	−0.0043 (17)	−0.0005 (17)
C18	0.078 (2)	0.0592 (18)	0.0782 (19)	0.0012 (15)	0.0042 (16)	−0.0011 (15)
N1	0.0693 (15)	0.0594 (13)	0.0599 (13)	−0.0137 (11)	−0.0038 (11)	0.0045 (10)
N2	0.0631 (15)	0.0628 (14)	0.0602 (13)	−0.0042 (11)	0.0047 (11)	−0.0009 (11)
O1	0.0685 (14)	0.0894 (15)	0.1015 (16)	−0.0033 (11)	−0.0311 (11)	−0.0102 (12)
O2	0.148 (2)	0.0899 (15)	0.0536 (12)	−0.0194 (13)	0.0032 (12)	0.0131 (11)
F1	0.1360 (18)	0.0734 (13)	0.154 (2)	−0.0270 (12)	0.0134 (14)	−0.0162 (12)
F2	0.0804 (14)	0.1292 (17)	0.1398 (18)	−0.0121 (11)	−0.0119 (12)	−0.0334 (13)
S1	0.0845 (6)	0.0688 (5)	0.0590 (5)	−0.0099 (4)	−0.0110 (4)	0.0051 (4)

### Geometric parameters (Å, º)

**Table:**

C1—C6	1.362 (4)	C10—H10A	0.9700
C1—C2	1.368 (4)	C10—H10B	0.9700
C1—C7	1.501 (4)	C11—N1	1.469 (3)
C2—C3	1.376 (4)	C11—H11A	0.9700
C2—H2	0.9300	C11—H11B	0.9700
C3—C4	1.363 (4)	C12—N2	1.457 (3)
C3—H3	0.9300	C12—C13	1.510 (4)
C4—C5	1.369 (4)	C12—H12A	0.9700
C4—S1	1.754 (3)	C12—H12B	0.9700
C5—C6	1.381 (4)	C13—C14	1.376 (4)
C5—H5	0.9300	C13—C18	1.377 (4)
C6—H6	0.9300	C14—C15	1.371 (4)
C7—H7A	0.9600	C14—H14	0.9300
C7—H7B	0.9600	C15—F1	1.346 (3)
C7—H7C	0.9600	C15—C16	1.359 (4)
C8—N1	1.472 (3)	C16—C17	1.352 (4)
C8—C9	1.508 (4)	C16—F2	1.356 (3)
C8—H8A	0.9700	C17—C18	1.377 (4)
C8—H8B	0.9700	C17—H17	0.9300
C9—N2	1.450 (3)	C18—H18	0.9300
C9—H9A	0.9700	N1—S1	1.632 (2)
C9—H9B	0.9700	O1—S1	1.432 (2)
C10—N2	1.457 (3)	O2—S1	1.419 (2)
C10—C11	1.497 (4)		
			
C6—C1—C2	116.7 (3)	N1—C11—C10	110.0 (2)
C6—C1—C7	121.2 (3)	N1—C11—H11A	109.7
C2—C1—C7	122.1 (3)	C10—C11—H11A	109.7
C1—C2—C3	122.8 (3)	N1—C11—H11B	109.7
C1—C2—H2	118.6	C10—C11—H11B	109.7
C3—C2—H2	118.6	H11A—C11—H11B	108.2
C4—C3—C2	119.5 (3)	N2—C12—C13	112.0 (2)
C4—C3—H3	120.3	N2—C12—H12A	109.2
C2—C3—H3	120.3	C13—C12—H12A	109.2
C3—C4—C5	118.9 (3)	N2—C12—H12B	109.2
C3—C4—S1	120.5 (2)	C13—C12—H12B	109.2
C5—C4—S1	120.5 (2)	H12A—C12—H12B	107.9
C4—C5—C6	120.4 (3)	C14—C13—C18	118.5 (3)
C4—C5—H5	119.8	C14—C13—C12	121.2 (3)
C6—C5—H5	119.8	C18—C13—C12	120.2 (3)
C1—C6—C5	121.7 (3)	C15—C14—C13	120.0 (3)
C1—C6—H6	119.2	C15—C14—H14	120.0
C5—C6—H6	119.2	C13—C14—H14	120.0
C1—C7—H7A	109.5	F1—C15—C16	119.2 (3)
C1—C7—H7B	109.5	F1—C15—C14	120.3 (3)
H7A—C7—H7B	109.5	C16—C15—C14	120.5 (3)
C1—C7—H7C	109.5	C17—C16—F2	120.3 (3)
H7A—C7—H7C	109.5	C17—C16—C15	120.6 (3)
H7B—C7—H7C	109.5	F2—C16—C15	119.1 (3)
N1—C8—C9	109.0 (2)	C16—C17—C18	119.3 (3)
N1—C8—H8A	109.9	C16—C17—H17	120.3
C9—C8—H8A	109.9	C18—C17—H17	120.3
N1—C8—H8B	109.9	C17—C18—C13	121.0 (3)
C9—C8—H8B	109.9	C17—C18—H18	119.5
H8A—C8—H8B	108.3	C13—C18—H18	119.5
N2—C9—C8	110.5 (2)	C11—N1—C8	111.8 (2)
N2—C9—H9A	109.6	C11—N1—S1	116.43 (17)
C8—C9—H9A	109.6	C8—N1—S1	118.03 (18)
N2—C9—H9B	109.6	C9—N2—C12	112.3 (2)
C8—C9—H9B	109.6	C9—N2—C10	109.7 (2)
H9A—C9—H9B	108.1	C12—N2—C10	110.5 (2)
N2—C10—C11	109.7 (2)	O2—S1—O1	120.16 (13)
N2—C10—H10A	109.7	O2—S1—N1	106.38 (12)
C11—C10—H10A	109.7	O1—S1—N1	106.30 (13)
N2—C10—H10B	109.7	O2—S1—C4	108.02 (14)
C11—C10—H10B	109.7	O1—S1—C4	107.81 (12)
H10A—C10—H10B	108.2	N1—S1—C4	107.58 (11)
			
C6—C1—C2—C3	1.5 (5)	C14—C13—C18—C17	−0.1 (4)
C7—C1—C2—C3	−178.0 (3)	C12—C13—C18—C17	177.0 (3)
C1—C2—C3—C4	−0.6 (4)	C10—C11—N1—C8	−56.5 (3)
C2—C3—C4—C5	−0.8 (4)	C10—C11—N1—S1	163.77 (18)
C2—C3—C4—S1	−179.4 (2)	C9—C8—N1—C11	55.7 (3)
C3—C4—C5—C6	1.2 (4)	C9—C8—N1—S1	−165.23 (18)
S1—C4—C5—C6	179.8 (2)	C8—C9—N2—C12	−175.5 (2)
C2—C1—C6—C5	−1.1 (5)	C8—C9—N2—C10	61.1 (3)
C7—C1—C6—C5	178.4 (3)	C13—C12—N2—C9	70.2 (3)
C4—C5—C6—C1	−0.2 (5)	C13—C12—N2—C10	−166.9 (2)
N1—C8—C9—N2	−57.8 (3)	C11—C10—N2—C9	−60.8 (3)
N2—C10—C11—N1	58.1 (3)	C11—C10—N2—C12	174.7 (2)
N2—C12—C13—C14	−129.4 (3)	C11—N1—S1—O2	−177.66 (19)
N2—C12—C13—C18	53.5 (4)	C8—N1—S1—O2	45.1 (2)
C18—C13—C14—C15	0.0 (4)	C11—N1—S1—O1	−48.5 (2)
C12—C13—C14—C15	−177.1 (3)	C8—N1—S1—O1	174.31 (19)
C13—C14—C15—F1	179.6 (3)	C11—N1—S1—C4	66.8 (2)
C13—C14—C15—C16	−0.3 (4)	C8—N1—S1—C4	−70.4 (2)
F1—C15—C16—C17	−179.3 (3)	C3—C4—S1—O2	−28.3 (3)
C14—C15—C16—C17	0.6 (5)	C5—C4—S1—O2	153.0 (2)
F1—C15—C16—F2	0.2 (4)	C3—C4—S1—O1	−159.6 (2)
C14—C15—C16—F2	−179.9 (3)	C5—C4—S1—O1	21.8 (3)
F2—C16—C17—C18	179.8 (3)	C3—C4—S1—N1	86.1 (2)
C15—C16—C17—C18	−0.7 (5)	C5—C4—S1—N1	−92.5 (2)
C16—C17—C18—C13	0.4 (5)		

### Hydrogen-bond geometry (Å, º)

**Table:**

*D*—H···*A*	*D*—H	H···*A*	*D*···*A*	*D*—H···*A*
C3—H3···O1^i^	0.93	2.67	3.380 (4)	134
C7—H7*A*···O1^ii^	0.96	2.66	3.400 (4)	134
C10—H10*B*···F1^iii^	0.97	2.66	3.585 (3)	160

Symmetry codes: (i) *x*−1, *y*, *z*; (ii) −*x*, −*y*+1, −*z*+2; (iii) *x*+1, −*y*+1/2, *z*+1/2.
